# Proprioceptive impairments in high fall risk older adults: the effect of mechanical calf vibration on postural balance

**DOI:** 10.1186/s12938-018-0482-8

**Published:** 2018-05-02

**Authors:** Nima Toosizadeh, Hossein Ehsani, Marco Miramonte, Jane Mohler

**Affiliations:** 1 Arizona Center on Aging (ACOA), Department of Medicine, College of Medicine, University of Arizona, Tucson, AZ 85724-5072 USA; 2 Division of Geriatrics, General Internal Medicine and Palliative Medicine, Department of Medicine, University of Arizona, Tucson, AZ USA; 3 Department of Biomedical Engineering, University of Arizona, Tucson, AZ USA

**Keywords:** Wearable motion sensors, Body sway, Mechanical stimulation, Geriatrics, Falling, Somatosensory system

## Abstract

**Background:**

Impairments in proprioceptive mechanism with aging has been observed and associated with fall risk. The purpose of the current study was to assess proprioceptive deficits among high fall risk individuals in comparison with healthy participants, when postural performance was disturbed using low-frequency mechanical gastrocnemius vibratory stimulation.

**Methods:**

Three groups of participants were recruited: healthy young (n = 10; age = 23 ± 2 years), healthy elders (n = 10; age = 73 ± 3 years), and high fall risk elders (n = 10; age = 84 ± 9 years). Eyes-open and eyes-closed upright standing balance performance was measured with no vibration, and 30 and 40 Hz vibration of both calves. Vibration-induced changes in balance behaviors, compared to baseline (no vibratory stimulation) were compared between three groups using multivariable repeated measures analysis of variance models.

**Results:**

Overall, similar results were observed for two vibration frequencies. However, changes in body sway due to vibration were more obvious within the eyes-closed condition, and in the medial–lateral direction. Within the eyes-closed condition high fall risk participants showed 83% less vibration-induced change in medial–lateral body sway, and 58% less sway velocity, when compared to healthy participants (*p *< 0.001; effect size = 0.45–0.64).

**Conclusions:**

The observed differences in vibration effects on balance performance may be explained by reduced sensitivity in peripheral nervous system among older adults with impaired balance.

## Background

Nearly 30% of older adults over 65 experience one or more falls every year, leading to hospitalization and death [1, 2]. The first step to prevent fall is to identify high fall risk individuals and understand the underlying mechanism that can lead to fall. Human balancing is a complex mechanism that involves several physiological systems, including sensory units (i.e., vestibular, visual, and proprioceptive), muscle reflexes, and the central nervous system [3, 4]. Any deterioration in components of this mechanism or interactions between components can compromise postural balance. As a result of aging, impairments in the proprioceptive component has been observed and associated with balance deficits [3, 5].

Within the current research, we studied proprioceptive deficits among high fall risk individuals in comparison with healthy participants when balance performance was disturbed using low-frequency mechanical calf vibration (gastrocnemius muscles). Among healthy adults, mechanical vibration of the gastrocnemius muscle increases the range and velocity of body sway and cause body tilt during upright standing [6, 7]. Previous work suggested that mechanical vibration can influence postural balance by activating the afferent nerves of muscle spindles, and increasing their firing rate [7], which can cause kinematic illusions [7, 8]. Although, several studies have focused on balance alterations due to mechanical vibrations, to our knowledge, no study has compared vibration-induced changes between healthy and high fall risk individuals. Further, balance behaviors among healthy participants have been influenced by the frequency of the vibration. Maximum effect of lateral gastrocnemius and tibialis anterior muscle vibration on postural balance has reported for high frequencies of 80–100 Hz among healthy individuals, which cause an increase in body sway [9]. On the other hand, vibration of plantar sole and triceps surae with very low frequencies of below 20 Hz reduces the body sway among healthy participants [10]. No study has investigated the effect of gastrocnemius muscle vibration on balance behaviors within frequencies near and slightly above the threshold for eliciting the postural balance.

We hypothesized that due to aging-induced alterations in muscle spindle sensory function, as well as decreased cortical and spinal excitability and slowed nerve conduction with aging [11], the vibratory stimulation, within a low frequency of 30 and 40 Hz, would have less influence on postural balance among high fall risk elders compared to healthy individuals. To confirm this, we assessed: (1) differences in balance behavior alterations due to vibration among three groups of healthy young, healthy older adults, and high fall risk older adults; and (2) the association between baseline balance performance and vibration-induced balance alterations.

## Methods

### Participants

Three groups of participants were recruited: healthy young adults (18–30 years), healthy older adults (≥ 65 years), and high fall risk older adults (≥ 65 years). High fall risk participants were selected according to the Center for Disease Control and Prevention’s STEADI Risk for Falling Assessment [12], which involves four questions, assigning one point to each affirmative response: (1) Have you fallen in the past year?; (2) Are you worried about falling?; (3) Do you feel unsteady when you are walking? (4) Have you had two or more falls? Those with a score of zero or one without a history of falling were considered low fall risk, and those with a score of two to four were considered high fall risk. Exclusion criteria for all three groups were: disorders associated with severe motor deficits and balance performance, including stroke, Parkinson’s disease, dementia (Mini-Mental State Examination (MMSE) score < 20) [13], severe arthritis in lower-extremities, cancer or diabetic neuropathy, vestibular diseases, and lower-extremity ulceration and amputation, history of dizziness, vertigo, and sedating medication or alcohol consumption within the prior 24 h. The above disorders were identified using subjective questionnaires as defined in previous work [14, 15], and participants were excluded if they claimed to have any related symptoms. Further, participants were excluded if they were not able to perform the initial practice balance trials as described below. For both healthy young and older adults, additional exclusion criterion of fall incident in a prior year was considered. All participants were recruited after completing written informed consent according to the principles expressed in the Declaration of Helsinki [16], approved by the University of Arizona’s Review Boards.

### Clinical measurements

Participants filled out clinical questionnaires before balance tests, including: (1) the visual analog pain scale for lower-extremity (VAS-10) (0: no pain–10: extreme pain) [17] within the prior 2-week period and at the time of the visit; (2) short falls efficacy scale-international (Short FES-I) for assessing the fear of falling [18]; and (3) the four-question fall scale (see above).

### Balance assessments

Each participant performed eight 30-s trials of balance assessment, including: two practice trials with no vibration system attached (one eyes-open and one eyes-closed), two trials with vibration system on calves but with no stimulation (one eyes-open and one eyes-closed), two trials with 30 Hz vibration (one eyes-open and one eyes-closed), and two trials with 40 Hz vibration (one eyes-open and one eyes-closed). Of note, data from practice trials were not used in analyses. In each trial, Romberg balance test was performed, during which, participants stood upright with their feet as close together as possible but without touching each other, and their arms crossed. For eyes-open trials no visual target was specified, and participants performed the balance facing a wall in 1-m distance from their standing point. The center-of-gravity (COG) was estimated using wearable motion sensors following identical procedures reported earlier [19–21]. Briefly, two sensors (one on the right shin and one on the back close to the lumbar), each including a tri-axial gyroscope, were used to estimate three-dimensional ankle and hip angles (BalanSens™; BioSensics, Boston, USA). A two-link inverted-pendulum model of the human body was used to calculate the COG from anterior–posterior and medial–lateral angles [19, 22]. The two-link model was used to calculate anterior–posterior (AP) and medial–lateral (ML) angles of legs (lower link-ankle rotation) and upper-body (upper link-hip rotation). Using participants’ anthropometric data, the mass and center of mass were estimated for each link [19]. A wavelet transform band-pass filter (Coiflet 5—cutoff frequency of 0.06–30 Hz) was used to reduce the noise related to skin movements [22].

Balance outcome measures included: ankle, hip, and COG overall sway and sway in anterior–posterior and medial–lateral directions, after excluding outliers as described in previous work [19]. Ankle, Hip, and COG sway in anterior–posterior or medial–lateral directions represent the range of sway in each direction [21]. Overall sway was estimated as the product of sway range in the anterior–posterior and medial–lateral directions. The range of sway for ankle and hip was represented as the angular range of motion in degrees for the anterior–posterior and medial–lateral sway, and in degrees^2^ for the overall sway. The COG range of sway was represented as the range of displacement in the anterior–posterior or the medial–lateral direction in centimeters, and in centimeters^2^ for the overall COG sway. Additional parameters included: sway velocity (overall COG sway distance divided by the test duration represented in centimeter/seconds), and body tilt in the anterior–posterior and medial–lateral directions (average COG location during the test duration in centimeters). Further, we calculated Romberg quotient (outcome measure within eyes-closed/outcome measure within eyes-open) for the above parameters to understand changes in balance parameters when visual feedback was removed [23]. For each balance parameter, percentage change was estimated comparing the stimulation condition with the no-stimulation condition.

### Vibration stimulation

Mechanical vibration of 30 and 40 Hz frequencies and 1 ± 0.002 mm amplitude were imposed to both gastrocnemius muscles. A pair of custom-made focal vibrator was used to generate mechanical stimulation using eccentric rotating servomotor. Velcro straps were used to attach the vibrators to the belly of the muscles. Previous studies showed that the postural effect with calf vibration, especially in high frequencies (60–90 Hz) increases after the onset of stimulation and saturates after ~ 30 min [6, 24]. Thus, here for balance trials with vibratory stimulations, participants were exposed to 1 min warm-up vibration prior to tests to assure effects of stimulation reach a plateau level. To minimize the residual effects of vibration on balance behaviors [6, 7], participants had a 2-min rest period between trials. Further, to minimize the residual effects of vibration, instead of randomizing the trials, balance with no vibration was performed first, followed by 30 and 40 Hz stimulation trials.

### Statistical analysis

Differences in demographic parameters among participant groups (balance groups: healthy young, healthy older adults, and high fall risk) were assessed using one-way analysis of variance (ANOVA) models. Differences in subjective questionnaires were assessed using multivariable ANOVA models, considering three balance groups, age, gender, and body mass index (BMI) as independent variables. To assess differences in balance behaviors between three balance groups, multivariable repeated measures ANOVA models were used. In each model, baseline balance parameters (trials with no stimulation) or percentage change in balance parameters due to vibration (compared to the condition with no stimulation) were considered as dependent variables; three balance groups, age, gender, vibration frequency (within subject variable), and BMI were considered as independent variables. Analyses were done separately for each of eyes-open and eyes-closed condition. Further, all data from eyes-open and eyes-closed conditions were combined and the analyses were repeated to investigate potential main effect of vision condition and interactions between vibration frequency and eyes-open/eyes-closed conditions. Cohen’s effect size was calculated for each ANOVA test, and post hoc Tukey’s honestly significant difference tests were performed for three pairwise comparison between the balance groups. The interaction effect between balance group and vibration frequency was also assessed.

Further analyses were performed to assess Pearson correlations (*r*) between baseline balance performance and changes in balance behaviors due to stimulation (without considering balance groups). Lastly, correlations between subjective questionnaires (i.e., the pain score, FES-I, and the fall score) and vibration-induce changes in balance parameters were assessed using linear regression models and reported as Pearson correlations. All analyses were done using JMP (Version 11, SAS Institute Inc., Cary, NC), and statistical significance was concluded when *p *< 0.05.

## Results

### Participants

Thirty participants were recruited, 10 healthy young adults, 10 healthy older adults, and 10 high fall risk older adults; mean (standard deviation-SD) age were 23 (2), 73 (3), and 84 (9) years, respectively. Demographic information and subjective questionnaires are reported in Table 1.

**Table 1:** Mean (standard deviation—SD or percentage) values of sociodemographic information and subjective questionnaires

	Healthy young	Healthy older adults	High fall risk	*p* value
Number, n (% of total)	10 (33%)	10 (33%)	10 (33%)	–
Male, n (% of the group)	5 (50%)	4 (40%)	3 (30%)	*χ*^*2*^(2) = 1.13; *p *= 0.57
Age, year (SD)	23.30 (2.26)	72.90 (2.81)	83.60 (9.46)	*F*(2,27) = 293.14; *p *< 0.0001*
Stature, cm (SD)	173.16 (9.66)	165.03 (10.91)	165.62 (11.21)	*F*(2,27) = 1.82; *p *= 0.18
Body mass, kg (SD)	70.84 (16.72)	64.71 (8.37)	65.24 (16.39)	*F*(2,27) = 0.56; *p *= 0.57
BMI, kg/m^2^	23.59 (4.81)	23.75 (2.11)	23.52 (4.08)	*F*(2,27) = 0.01; *p *= 0.99
Pain at the moment, 0–10 (SD)	0 (0)	0.20 (0.63)	1.90 (2.69)	*F*(2,24) = 1.98; *p *= 0.16
Pain within 2 weeks, 0–10 (SD)	0.10 (0.32)	0.80 (2.53)	3.50 (3.72)	*F*(2,24) = 2.02; *p *= 0.15
Short FES-I, 7–28 (SD)	7.30 (0.64)	8.00 (1.63)	14.90 (3.96)	*F*(2,24) = 14.66; *p *< 0.0001*
Fall score, 0–4 (SD)	0.00 (0)	0.10 (0.32)	3.10 (0.74)	*F*(2,24) = 48.42; *p *< 0.0001*
Number of falls within 1 year(SD)	0.00 (0)	0.00 (0)	2.88 (4.64)	*F*(2,24) = 7.43; *p *< 0.001*

*BMI* body mass index, *FES-I* falls efficacy scale-international

* Significant *p* value

### Balance behaviors among healthy and high fall risk

Baseline balance behaviors (without stimulation) were different among three groups. Larger ankle, hip, and COG sway were observed among high fall risk older adults compared to healthy participants. These differences were significant only for the eyes-closed condition (Table 2); none of baseline balance parameters were significantly different between groups when participants performed the test with eyes open (*p *> 0.06). Differences in baseline balance parameters among high fall risk participants and healthy groups (young and older adults) were more noticeable for medial–lateral compared to anterior–posterior sway. Post hoc Tukey’s tests showed no significant difference among baseline balance parameters for pairwise comparisons between balance groups. None of the baseline parameters representing anterior–posterior and medial–lateral body tilt were significantly different between groups within eyes-open or eyes-closed conditions (*p *> 0.11).

**Table 2:** Mean (standard deviation) values of baseline balance parameters (no vibration) between balance groups

Eyes-open	Healthy young	Healthy older adults	High fall risk	*p* value^a^	Effect size
Ankle sway					
Medial–lateral, deg	1.21 (0.55)	1.38 (0.60)	1.69 (0.80)	*F*(2,24) = 2.14; *p *= 0.14	0.31
Anterior–posterior, deg	1.64 (0.66)	1.65 (0.66)	2.07 (1.03)	*F*(2,24) = 2.37; *p *= 0.12	0.26
Overall, deg^2^	1.82 (1.23)	2.04 (1.31)	2.84 (2.56)	*F*(2,24) = 2.81; *p *= 0.08	0.26
Hip sway					
Medial–lateral, deg	1.09 (0.37)	1.00 (0.20)	1.31 (0.38)	*F*(2,24) = 2.16; *p *= 0.14	0.41
Anterior–posterior, deg	2.40 (1.22)	1.94 (0.76)	3.20 (1.73)	*F*(2,24) = 2.54; *p *= 0.10	0.42
Overall, deg^2^	2.26 (1.92)	1.61 (1.03)	3.17 (1.88)	*F*(2,24) = 2.02; *p *= 0.15	0.40
COG sway					
Medial–lateral, cm	0.48 (0.21)	0.53 (0.19)	0.64 (0.30)	*F*(2,24) = 2.58; *p *= 0.10	0.29
Anterior–posterior, cm	0.89 (0.29)	0.93 (0.30)	1.08 (0.42)	*F*(2,24) = 1.11; *p *= 0.35	0.24
Overall, cm^2^	0.40 (0.27)	0.40 (0.23)	0.58 (0.43)	*F*(2,24) = 2.76; *p *= 0.08	0.27
Sway velocity, cm/s	0.66 (0.09)	0.84 (0.22)	0.84 (0.26)	*F*(2,24) = 2.88; *p *= 0.06	0.45

Eyes-closed	Healthy young	Healthy older adults	High fall risk	*p* value^a^	Effect size
Ankle sway					
Medial–lateral, deg	1.70 (0.58)	1.36 (0.33)	2.23 (0.76)	*F*(2,24) = 5.01; *p *= 0.02*	0.64
Anterior–posterior, deg	1.83 (0.93)	2.07 (0.91)	2.45 (0.62)	*F*(2,24) = 1.34; *p *= 0.28	0.31
Overall, deg^2^	2.62 (1.44)	2.49 (1.75)	3.93 (1.96)	*F*(2,24) = 2.87; *p *= 0.08	0.34
Hip sway					
Medial–lateral, deg	1.63 (0.42)	1.40 (0.42)	1.82 (0.53)	*F*(2,24) = 5.65; *p *< 0.01*	0.37
Anterior–posterior, deg	2.64 (0.90)	2.73 (1.01)	4.22 (1.16)	*F*(2,24) = 5.57; *p *= 0.01*	0.71
Overall, deg^2^	3.25 (1.52)	2.94 (1.73)	6.53 (4.09)	*F*(2,24) = 2.87; *p *< 0.01*	0.67
COG sway					
Medial–lateral, cm	0.76 (0.27)	0.55 (0.14)	0.90 (0.32)	*F*(2,24) = 4.41; *p *= 0.02*	0.60
Anterior–posterior, cm	1.05 (0.39)	1.17 (0.51)	1.46 (0.39)	*F*(2,24) = 1.37; *p *= 0.27	0.40
Overall, cm^2^	0.65 (0.33)	0.54 (0.33)	1.04 (0.59)	*F*(2,24) = 3.40; *p *= 0.05*	0.51
Sway velocity, cm/s	0.89 (0.23)	0.72 (0.10)	0.95 (0.26)	*F*(2,24) = 3.52; *p *= 0.04*	0.49

*COG* center of gravity

* Significant *p* value

^a^ANOVA models were adjusted for age, gender, and body mass index

**Fig. 1:**
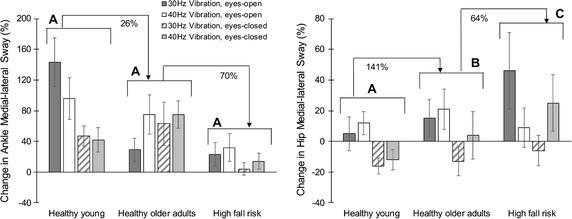
Mean (standard error) changes in the ankle and hip medial–lateral sway due to vibration. Differences in balance alteration are presented between three groups of healthy young, healthy older adults, and high fall risk older adults. Post hoc Tukey’s test grouping is presented using bold letters

Alterations in balance behaviors were observed when the vibratory stimulation was applied to calves. Similar to baseline balance behaviors, between group differences in body sway changes due to vibration were more obvious within the eyes-closed condition and in the medial–lateral direction (Table 3). Overall, it was noticeable that high fall risk older adults showed less changes in COG sway in response to vibratory stimulation compared to the healthy control groups. Specifically, on average in the eyes-closed condition within balance trials with vibration, 5% increase in medial–lateral and 3% increase in anterior–posterior COG sways were observed in the high fall risk group compared to baseline trials; corresponding changes were 46 and 30% in medial–lateral and anterior–posterior directions for healthy older adults, and 20 and 59% for healthy young participants. Within the medial–lateral direction (and within the anterior–posterior direction although not significant), a decreasing trend in ankle sway alterations was seen from healthy young and healthy older adults to high fall risk groups (Fig. 1 and Table 3). On the other hand, hip sway in the medial–lateral direction demonstrated an increasing trend from healthy young and older adult groups to the high fall risk group (Fig. 1 and Table 3). According to these results, when exposed to vibratory stimulation, high fall risk older adults showed smaller ankle sway and larger hip sway changes compared to healthy groups. Also, changes in sway velocity were significantly different between groups; on average high fall risk participants showed 42 and 58% less changes in sway velocity in response to vibration, compared to other groups within eyes-open and eyes-closed conditions (Table 3). Post hoc Tukey’s tests showed significant pairwise differences between healthy young and healthy older adult groups, as well as between healthy older adults and high fall risk groups for medial–lateral hip sway changes within both eyes-open and eyes-closed conditions (Fig. 1). No significant difference in body tilt changes due to vibration was observed between three groups within eyes-open or eyes-closed conditions (*p *> 0.14). Also, results from Romberg quotient showed no significant group effect in changes in parameters within the eyes-closed condition compared to the eyes-open condition (*p *> 0.06).

**Table 3:** Mean (standard deviation) values of the percentage change in balance parameters in three balance groups

Eyes-open	Healthy young	Healthy older adults	High fall risk	*p* value^a^	Effect size
Ankle sway					
Medial–lateral 30 Hz, %	143 (100)	29 (48)	23 (49)	*F*(2,51) = 0.14; *p *= 0.87	0.56
Medial–lateral 40 Hz, %	96 (86)	75 (80)	32 (57)		
Anterior–posterior 30 Hz, %	73 (96)	50 (73)	22 (49)	*F*(2,51) = 0.48; *p *= 0.62	0.47
Anterior–posterior 40 Hz, %	110 (84)	44 (39)	14 (45)		
Overall 30 Hz, %	95 (526)	85 (121)	23 (59)	*F*(2,51) = 0.20; *p *= 0.82	0.68
Overall 40 Hz, %	305 (263)	94 (126)	50 (113)		
Hip sway					
Medial–lateral 30 Hz, %	5 (35)	15 (38)	46 (79)	*F*(2,51) = 3.49; *p *= 0.03*	0.18
Medial–lateral 40 Hz, %	12 (24)	21 (42)	9 (40)		
Anterior–posterior 30 Hz, %	32 (53)	32 (38)	37 (77)	*F*(2,51) = 1.04; *p *= 0.36	0.31
Anterior–posterior 40 Hz, %	76 (78)	46 (115)	3 (51)		
Overall 30 Hz, %	38 (71)	73 (111)	110 (157)	*F*(2,51) = 1.25; *p *= 0.30	0.08
Overall 40 Hz, %	120 (117)	44 (80)	15 (86)		
COG sway					
Medial–lateral 30 Hz, %	109 (68)	23 (37)	24 (38)	*F*(2,51) = 0.14; *p *= 0.87	0.57
Medial–lateral 40 Hz, %	118 (132)	54 (74)	29 (51)		
Anterior–posterior 30 Hz, %	72 (102)	36 (45)	28 (55)	*F*(2,51) = 0.25; *p *= 0.78	0.36
Anterior–posterior 40 Hz, %	76 (74)	46 (62)	11 (42)		
Overall 30 Hz, %	391 (653)	61 (53)	59 (120)	*F*(2,51) = 0.53; *p *= 0.59	0.56
Overall 40 Hz, %	194 (181)	67 (92)	40 (103)		
Sway velocity 30 Hz, %	158 (74)	188 (154)	123 (93)	*F*(2,51) = 4.12; *p *= 0.02*	0.38
Sway velocity 40 Hz, %	193 (163)	225 (89)	100 (69)		

Eyes-closed	Healthy young	Healthy older adults	High fall risk	*p* value^a^	Effect size
Ankle sway					
Medial–lateral 30 Hz, %	47 (42)	63 (89)	4 (25)	*F*(2,51) = 7.80; *p *< 0.01*	0.50
Medial–lateral 40 Hz, %	42 (51)	75 (57)	14 (33)		
Anterior–posterior 30 Hz, %	71 (78)	20 (47)	− 9 (12)	*F*(2,51) = 1.39; *p *= 0.26	0.66
Anterior–posterior 40 Hz, %	98 (73)	52 (90)	1 (28)		
Overall 30 Hz, %	165 (213)	82 (176)	− 4 (34)	*F*(2,51) = 1.93; *p *= 0.16	0.54
Overall 40 Hz, %	180 (145)	113 (132)	17 (59)		
Hip sway					
Medial–lateral 30 Hz, %	− 16 (16)	− 13 (30)	− 6 (31)	*F*(2,51) = 21.43; *p *< 0.0001*	0.28
Medial–lateral 40 Hz, %	− 12 (21)	4 (49)	25 (58)		
Anterior–posterior 30 Hz, %	22 (38)	11 (66)	6 (43)	*F*(2,51) = 0.54; *p *= 0.59	0.10
Anterior–posterior 40 Hz, %	− 12 (21)	4 (49)	25 (58)		
Overall 30 Hz, %	− 08 (44)	9 (40)	13 (81)	*F*(2,51) = 9.50; *p *< 0.001*	0.17
Overall 40 Hz, %	14 (47)	21 (75)	48 (110)		
COG sway					
Medial–lateral 30 Hz, %	22 (30)	46 (72)	− 2 (18)	*F*(2,51) = 9.20; *p *< 0.001*	0.45
Medial–lateral 40 Hz, %	18 (34)	47 (45)	13 (30)		
Anterior–posterior 30 Hz, %	57 (84)	20 (53)	− 3 (30)	*F*(2,51) = 0.78; *p *= 0.46	0.41
Anterior–posterior 40 Hz, %	61 (65)	40 (66)	9 (38)		
Overall 30 Hz, %	93 (133)	67 (168)	− 5 (30)	*F*(2,51) = 2.12; *p *= 0.13	0.34
Overall 40 Hz, %	86 (100)	59 (100)	24 (60)		
Sway velocity 30 Hz, %	108 (53)	162 (92)	60 (39)	*F*(2,51) = 8.89; *p *< 0.001*	0.64
Sway velocity 40 Hz, %	139 (82)	173 (109)	61 (43)		

*COG* center of gravity

* Significant *p* value

^a^ANOVA models were adjusted for age, gender, and body mass index

A significant main effect of vibration frequency was observed only in hip medial–lateral sway changes within the eyes-closed condition (*p *= 0.03); hip medial–lateral sway increased among all three groups when exposed to 40 Hz vibration (5% change), and it decreased when exposed to 30 Hz vibration (− 12% change). However, this difference was not observed in any other parameter within eyes-open or eyes-closed conditions (*p *> 0.15). Further, significant main effect of vision condition (eyes-open versus eyes-closed) was observed in vibration-induced changes in ankle and COG medial–lateral sway, and hip medial–lateral, anterior–posterior, and overall sway (*p *< 0.01); in all conditions larger amount of sway changes was observed within the eyes-closed condition. No significant interaction effect of balance groups and vibration frequency or vibration frequency and eyes-open/eyes-closed conditions on balance parameters was observed (*p *> 0.07).

### Association between baseline data and balance alterations

**Fig. 2:**
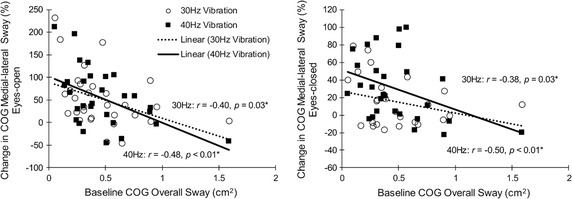
Correlations between changes in COG sway in medial–lateral direction and baseline overall COG sway. All participants from healthy young, healthy older adults, and high fall risk older adults’ groups were included in the analysis

Changes in ankle and COG sway due to vibration, especially in the medial–lateral direction, were negatively correlated with baseline overall COG sway (*r *= 37–0.58; *p *< 0.04); participants with higher baseline sway showed smaller changes (Fig. 2). Further, significant negative correlations were observed between fall score and changes in anterior–posterior ankle sway due to vibration among all conditions except for the eyes-open 30 Hz condition (*r *> 0.41 and *p *< 0.03). No other significant correlation was observed between other questionnaires and changes in balance parameters (*p *> 0.05).

## Discussion

### Vibration effects on balance

As hypothesized, vibratory stimulation influenced balance behaviors among high fall risk individuals differently compared to healthy young and older adults. Increase in the overall amount and velocity of body sway was observed as a result of calf vibration among participants. The increase was minimum for high fall risk participants. In addition to overall body sway, differences in balancing strategies (ankle versus hip sway) were also observed among three groups. Focusing on the ankle joint, increase in sway range due to vibration was smaller among the high fall risk group compared to healthy groups. Two explanations have been suggested previously as potential reasons for the increased body sway due to vibration among healthy individuals. Previous knowledge was expanded here to explain observed differences between high fall risk older adults and healthy individuals.

The first explanation is that changes in postural muscle activities due to vibration can increase the range and speed of body sway during upright standing by affecting the peripheral nervous system and short-latency reflexes. Specifically for the ankle joint, small angular deviations continuously occur during upright standing, which can lead to changes in lengths of lower-extremity muscles and muscle spindle activation [25]. Signals from muscle spindles are directed to motor neurons, which activate the parent muscles to restore joint position [25]. Mechanical vibration of a muscle fascicle can excite spindles and increase the muscle firing rate [26]. Increase in muscle activity leads to shortening of the excited muscles and simultaneously co-activity of antagonist muscles [7], and consequently affect the interaction between the sensory spindles and the muscle motor executive system. With aging, the efficiency of this reflexive loop declines due to aging of muscle spindles. Previous studies suggested that deterioration in muscle spindle performance with aging happens because of changes in covering capsule dimensions, reduced number of intrafusal fibers within spindles, and denervation process [11]. Similar to findings here, a smaller effect of muscle tendon vibration on dynamic position sensing of the ankle joint has been reported among elderly compared to young healthy participants [27].

The second explanation is related to vibration-induced alterations in the central nervous system performance and long-latency responses. Proprioceptive feedback from muscle spindles, in addition to providing data for the local reflexive regulation, provides information for the central nervous system regarding the level of motor activities [28]. To maintain balance, these information are used in the brain cortex to adjust muscle activities [29]. Vibration of muscle can cause some illusionary sensation in the brain regarding the lower-extremity position [8, 11]. Previous studies showed deterioration in position and motion sensing of muscle spindles with aging [11, 30], and aging-induced alterations in central nervous system, such as decreased attentional resources and a general loss of neural substrate [31, 32]. Therefore, it has been hypothesized here that vibration would cause less illusionary disturbance within central nervous system among elders with impaired balance since messages from spindle units are weaker and the central nervous system may be less sensitive to the disturbance of these messages.

Unlike ankle sway, overall, high fall risk participants showed larger increase in hip sway within the eyes-closed condition compared to healthy groups (Table 3). As a result of neuromuscular complications, weakness, and limitations in the ankle joint motion, larger compensatory motion from the hip and trunk is required to correct the posture during upright standing among high fall risk elders [5, 20]. This may have happened because the former group tend to implement hip-strategy (proximal-to-distal sequencing of muscle activation) more commonly for maintaining balance. Interestingly, similar to findings here, Manchester et al. [33] reported a higher tendency in using hip for maintaining balance among somatosensory impaired older adults compared to healthy young participants when ankle somatosensation was limited.

Findings here suggest that differences in balance performance between three groups were more detectable within the eyes-closed condition. This is in agreement with previous studies showing that age-related deficits in balance performance and alterations in balance behaviors due to vibration were manifested without visual input [6, 34]. Further within current results, body sway in the medial–lateral direction could better demonstrate differences in balance behaviors between high fall risk and healthy participants compared to the anterior–posterior direction. This may happen due to different balance control mechanisms in different directions. Flexor and extensor lower-extremity muscles provide moments in a similar direction to control anterior–posterior body sway [4, 20]. On the other hand, invertor and evertor lower-extremity muscles help in maintaining balance in the medial–lateral direction implementing a cancellation strategy [4, 20]. More investigation is required to understand the association between vibratory stimulation and changes in balance mechanisms.

Regardless of balance grouping, we observed negative correlations between baseline balance performance and vibration-induced changes in balance. Those participants that showed poor baseline balance performance, were more likely to show less influence from the vibration stimulation. In agreement to our findings for between-group differences, poor sensory performance may result in poor baseline balance performance, as well as less sensation of vibration and accordingly less vibration-induced changes in balance among individuals. However, more accurate measurements of deficits within balancing mechanism are required to confirm this hypothesis.

### Limitations and future direction

Previous research suggested that vibration frequency and amplitude affect body sway during upright standing [35]. One limitation of the current study is the lack of additional testing conditions for different vibration frequencies (higher frequencies above 40 Hz) and amplitudes. Within the current study lower frequencies of vibration were used, because high vibratory stimulations (60–90 Hz) have been associated with longer residual effects [6, 24]. Also, we only focused on gastrocnemius muscle vibration, and therefore, conclusions here may not be generalized for other lower-extremity muscle vibrations.

Further, high fall risk participants were selected based on the history of fall and poor balance. Therefore, any conclusion regarding the association between the type of balance deterioration (peripheral versus central nervous system) and vibratory stimulation requires further investigation, recruiting participants with pre-diagnosed balance disorders. Also, current findings cannot confirm lack of sensory performance deficits among low fall risk participants, as we only compared them with high fall risk elders, without any direct measurement of sensory performance. Further, significant differences in age exist between selected groups. Although all between group comparisons were adjusted with age here, investigating balance differences due to vibration among older adults with similar age ranges worth future investigations.

Finally, although findings were encouraging they should be interpreted cautiously, due to the lack of direct testing of proprioceptive alterations in response to vibration. Specifically, the accuracy of mechanical vibration as a testing tool for assessing high fall risk older adults should be confirmed using direct measures of deficits in peripheral (as well as central) nervous system.

### Clinical implications

Due to ease-of-use, mechanical vibratory stimulation on calves can potentially be implemented in clinical settings for assessing older adults with impaired ankle proprioceptive performance and high fall risk. Unlike subjective evaluation of peripheral sensation, measuring body sway using body-worn motion sensors has the advantage of providing an objective scale of balance deficits due to ankle proprioceptive impairments, which can be directly related to fall risk.

Further, more than half of participants from the high fall risk group showed improvements in balance (reduced overall body sway compared to baseline) when they were exposed to 30 Hz mechanical vibration. Interestingly, less than 10% of healthy young or elderly participants showed smaller overall body sway after vibration. These improvements in balance may happen due to activation of muscle proprioceptors as a result of vibration. Hypothetically, mechanical vibration can increase the excitability of muscle motor neurons by adding a stochastic resonance noise to sensory signals and reducing the muscle activation threshold. This hypothesis has been supported by the data on enhancement of the muscle spindle sensitivity and postural balance among elders using electrical noise signal stimulation [36]. Associations between mechanical calf vibration, vibration frequency and amplitude, and vibration duration with balance improvements are left to be studied in future.

## Conclusions

Within the current study the mechanical vibration was used to impact the performance of proprioceptive system during upright balance standing. Although the effect of vibratory stimulation on balance performance among healthy individuals has been studied before, for the first time, we tested vibratory stimulation to compare balance behaviors between high fall risk older adults and healthy young and elderly participants. We observed that changes in ankle and overall body sway were significantly smaller in the high fall risk group compared to heathy groups. On the other hand, the high fall risk group showed larger hip sway for maintaining balance when imposed to vibratory stimulation compared to healthy individuals. These changes were more prominent during eyes-closed condition and in the medial–lateral direction. The observed differences in vibration effects may be explained by reduced sensitivity in peripheral and central nervous system in older adults with impaired balance.

## Abbreviations

VAS: visual analog scale
FES-I: falls efficacy scale-international
COG: center of gravity
ANOVA: analysis of variance
BMI: body mass index
SD: standard deviation

## References

[1] Liu-Ambrose T, Davis JC, Hsu CL, Gomez C, Vertes K, Marra C, Brasher PM, Dao E, Khan KM, Cook W (2015). Action Seniors!-secondary falls prevention in community-dwelling senior fallers: study protocol for a randomized controlled trial. Trials.

[2] Mohler MJ, Wendel CS, Taylor-Piliae RE, Toosizadeh N, Najafi B (2016). Motor performance and physical activity as predictors of prospective falls in community-dwelling older adults by frailty level: application of wearable technology. Gerontology.

[3] Horak FB (2006). Postural orientation and equilibrium: what do we need to know about neural control of balance to prevent falls?. Age Ageing.

[4] Winter DA (1995). Human balance and posture control during standing and walking. Gait Posture.

[5] Horak FB, Shupert CL, Mirka A (1989). Components of postural dyscontrol in the elderly: a review. Neurobiol Aging.

[6] Capicikova N, Rocchi L, Hlavacka F, Chiari L, Cappello A (2006). Human postural response to lower leg muscle vibration of different duration. Physiol Res.

[7] Wierzbicka M, Gilhodes J, Roll J (1998). Vibration-induced postural posteffects. J Neurophysiol.

[8] Roll J, Vedel J, Ribot E (1989). Alteration of proprioceptive messages induced by tendon vibration in man: a microneurographic study. Exp Brain Res.

[9] Polonyova A, Hlavacka F (2001). Human postural responses to different frequency vibrations of lower leg muscles. Physiol Res.

[10] Naka M, Fujiwara K, Kiyota N (2015). Postural responses to various frequencies of vibration of the triceps surae and forefoot sole during quiet standing. Perception.

[11] Goble DJ, Coxon JP, Wenderoth N, Van Impe A, Swinnen SP (2009). Proprioceptive sensibility in the elderly: degeneration, functional consequences and plastic-adaptive processes. Neurosci Biobehav Rev.

[12] Rubenstein LZ, Vivrette R, Harker JO, Stevens JA, Kramer BJ (2011). Validating an evidence-based, self-rated fall risk questionnaire (FRQ) for older adults. J Saf Res.

[13] Folstein MF, Folstein SE, McHugh PR (1975). “Mini-mental state”: a practical method for grading the cognitive state of patients for the clinician. J Psychiatr Res.

[14] Speechley M, Tinetti M (1991). Falls and injuries in frail and vigorous community elderly persons. J Am Geriatr Soc.

[15] Myers A, Young Y, Langlois J (1996). Prevention of falls in the elderly. Bone.

[16] Association WM (2013). World Medical Association Declaration of Helsinki: ethical principles for medical research involving human subjects. JAMA.

[17] Langley G, Sheppeard H (1985). The visual analogue scale: its use in pain measurement. Rheumatol Int.

[18] Kempen GI, Yardley L, Van Haastregt JC, Zijlstra GR, Beyer N, Hauer K, Todd C (2008). The Short FES-I: a shortened version of the falls efficacy scale-international to assess fear of falling. Age Ageing.

[19] Najafi B, Horn D, Marclay S, Crews RT, Wu S, Wrobel JS (2010). Assessing postural control and postural control strategy in diabetes patients using innovative and wearable technology. J Diabetes Sci Technol.

[20] Toosizadeh N, Lei H, Schwenk M, Sherman SJ, Sternberg E, Mohler J, Najafi B (2014). Does integrative medicine enhance balance in aging adults? Proof of concept for the benefit of electroacupuncture therapy in Parkinson’s disease. Gerontology.

[21] Toosizadeh N, Mohler J, Lei H, Parvaneh S, Sherman S, Najafi B (2015). Motor performance assessment in Parkinson’s disease: association between objective in-clinic, objective in-home, and subjective/semi-objective measures. PLoS ONE.

[22] Toosizadeh N, Mohler J, Armstrong DG, Talal TK, Najafi B (2015). The influence of diabetic peripheral neuropathy on local postural muscle and central sensory feedback balance control. PLoS ONE.

[23] Howcroft J, Lemaire ED, Kofman J, McIlroy WE (2017). Elderly fall risk prediction using static posturography. PLoS ONE.

[24] Tjernström F, Fransson P-A, Hafström A, Magnusson M (2002). Adaptation of postural control to perturbations—a process that initiates long-term motor memory. Gait Posture.

[25] Horak FB, Nashner LM (1986). Central programming of postural movements: adaptation to altered support-surface configurations. J Neurophysiol.

[26] Burke D, Hagbarth K-E, Löfstedt L, Wallin BG (1976). The responses of human muscle spindle endings to vibration of non-contracting muscles. J Physiol.

[27] Verschueren S, Brumagne S, Swinnen S, Cordo P (2002). The effect of aging on dynamic position sense at the ankle. Behav Brain Res.

[28] Hulliger M (1984). The mammalian muscle spindle and its central control. Reviews of physiology, biochemistry and pharmacology.

[29] Mihara M, Miyai I, Hatakenaka M, Kubota K, Sakoda S (2008). Role of the prefrontal cortex in human balance control. Neuroimage.

[30] Liu J-X, Eriksson P-O, Thornell L-E, Pedrosa-Domellöf F (2005). Fiber content and myosin heavy chain composition of muscle spindles in aged human biceps brachii. J Histochem Cytochem.

[31] Raz N, Rodrigue KM (2006). Differential aging of the brain: patterns, cognitive correlates and modifiers. Neurosci Biobehav Rev.

[32] Toosizadeh N, Najafi B, Reiman EM, Mager RM, Veldhuizen JK, O’Connor K, Zamrini E, Mohler J (2016). Upper-extremity dual-task function: an innovative method to assess cognitive impairment in older adults. Front Aging Neurosci.

[33] Manchester D, Woollacott M, Zederbauer-Hylton N, Marin O (1989). Visual, vestibular and somatosensory contributions to balance control in the older adult. J Gerontol.

[34] Smetanin B, Popov K, Kozhina G (2004). Specific and nonspecific visual influences on the stability of the vertical posture in humans. Neurophysiology.

[35] Abrahámová D, Mancini M, Hlavačka F, Chiari L (2009). The age-related changes of trunk responses to Achilles tendon vibration. Neurosci Lett.

[36] Gravelle DC, Laughton CA, Dhruv NT, Katdare KD, Niemi JB, Lipsitz LA, Collins JJ (2002). Noise-enhanced balance control in older adults. NeuroReport.

